# Fabrication of pH-responsive PLGA(UCNPs/DOX) nanocapsules with upconversion luminescence for drug delivery

**DOI:** 10.1038/s41598-017-16948-4

**Published:** 2017-12-21

**Authors:** Junwei Zhao, Hui Yang, Jili Li, Yujiang Wang, Xin Wang

**Affiliations:** 1 Materials Science and Engineering School, Luoyang Institute of Science and Technology, Luoyang, 471023 P. R. China; 2 Division of Nanobiomedicine, Suzhou Institute of Nano-Tech and Nano-Bionics, Chinese Academy of Sciences, Suzhou, 215123 P. R. China

## Abstract

The integration of anticancer drugs and inorganic nanocrystals in polymer nanocapsules is a widely used strategy to improve their functionality, stability and sustained release. However, the complexity in the preparation of functional nanocapsules and their reproducibility still challenge these promising drug carriers in clinical application. Here we introduce a simple one-step self-assembly strategy to prepare multifunctional nanocapsules based on simultaneous poly (DL-lactic-co-glycolic acid) (PLGA) encapsulation of antitumor drug doxorubicin hydrochloride (DOX) and NaYF_4_:Yb,Er@NaGdF_4_ upconversion nanoparticles (UCNPs) for cancer cell imaging and drug delivery. The obtained PLGA(UCNPs/DOX) nanocapsules with a small size of ≈150 nm possessed bright upconversion fluorescence and could act as *T*_1*-*_weighted contrast agents for magnetic resonance imaging (MRI). Moreover, the PLGA(UCNPs/DOX) nanocapsules exhibited pH-responsive drug releasing behavior, causing the loaded DOX easily releasing at cancer cells, and an obvious cytotoxicity *via* MTT assay. The endocytosis process of PLGA (UCNPs/DOX) nanocapsules is evaluated using optical microscopy and upconversion fluorescence microscopy. These results demonstrated that the developed PLGA nanocapsules could serve as multifunctional drug delivery systems for cancer imaging and therapy.

## Introduction

Recently, multifunctional nanocomposites that would simultaneously possess diagnosis, target and therapy function have attracted much attention for cancer treatment and tumor suppression^1^. The design of specific stimuli-responsive systems is prospective since the anticancer drugs are stable during delivery and may be released at the targeted cells in response to external stimuli such as temperature, light irradiation, redox reagents, pH, enzymes, and ionic strength^2–11^. Among these “smart” carriers, a pH-responsive system for encapsulating anti-tumor drugs has been a hot research topic in view of the fact that the interstitial fluids of many solid tumors have lower pH values in contrast to the surrounding normal tissue^12–14^. Over the past few decades, numerous stimuli-responsive drug delivery systems have been developed as multifunctional nanocapsules, which are able to specifically accumulate in the required organ or tissue and then penetrate inside target cells, releasing the drugs^6–8^. Therefore, many strategies have been developed to fabricate smart polymeric materials as drug carriers, which are capable of responding to a great diversity of external triggers and enhance the therapeutic efficiency of anticancer drugs by facilitating local drug uptake^9,15^. Among these systems, poly (DL-lactic-co-glycolic acid) (PLGA), approved by the US Food and Drug Administration (FDA) and European Medicine Agency (EMA)^16,17^, is a relatively ideal choice polymer because of their excellent biocompatibility and tunable biodegradability. Besides, PLGA nanoparticles (NPs) also exhibit a high loading capacity of various insoluble therapeutics^11^. In the previous reports, the effectiveness of PLGA NPs as nanocarriers has been established for the encapsulation of poor water-soluble drugs, such as paclitaxel^18^, haloperidol^19^, and estradiol^10^.

Luminescent inorganic NPs have attracted immense attention in the past decade because of their potential application in biolabeling, sensing, bioimaging, and clinical therapeutics^20–26^. In particular, lanthanide-doped upconversion nanoparticles (UCNPs), which are able to convert NIR excitation into shorter-wavelength emissions, are recognized as excellent biomedical detection and diagnostic materials because of their unique features such as high photochemical stability, sharp emission bandwidth, and large anti-Stokes shift^20,24^. As reported in the previous work^25^, lanthanide-doped UCNPs can not only act as fluorescence imaging agents for cancer diagnosis, but also *T*_1_-weighted contrast agents for magnetic resonance imaging (MRI) owing to the existence of paramagnetic gadolinium ions (Gd^3+^)^21,23,27–29^. In the past years, many multifunctional composites have been prepared for the simultaneous diagnosis and treatment of cancer by combining fluorescence imaging materials and pharmacologically active drug^4,5,22^. Zhang *et al*. synthesized nanorattles composed of a magnetic core and an upconversion luminescent shell, encapsulating the anticancer drug doxorubicin (DOX) using the free volume of the composites^30^. Graphene oxide as a drug nanocarrier was introduced to carry DOX, camptothecin and rhodamine^31^. A multifunctional polymer was designed as biomedical nano-platform to provide effective targeting to folate receptors, detection by MRI and fluorescence imaging, and cell growth inhibition in KB cancer cells^32^. Shi *et al*. developed a multifunctional nanocarrier system for cell targeting, and anticancer drugs for localized treatment^33^. A drug delivery system was developed based on PS-*b*-PAA capped NaYF_4_:Yb,Er UCNPs, superparamagnetic NCs and DOX^34^. Nevertheless, most of multifunctional nanocomposites often suffer from complex synthetic processes, lack of size-control, and limited therapeutic efficiency of cancer.

In this work, we developed a simple one-step self-assembly strategy to prepare multifunctional PLGA nanocapsules, which incorporated simultaneously with anti-tumor drug DOX and NaYF_4_:Yb,Er@NaGdF_4_ UCNPs as MRI and fluorescence imaging agents, for bioimaging and drug delivery. The DOX could serve as a dual-functional agent with integrated chemotherapy and optical imaging probes capabilities. Meanwhile, the NaYF_4_:Yb,Er@NaGdF_4_ NPs could serve as a dual-model imaging contrast agent for upconversion fluorescence and *T*_1_-weighted MRI. Therefore, the fluorescence of DOX or UCNPs in PLGA nanocapsules can be monitored to demonstrate the cellar localization and internalization. The pH-response release behavior of DOX in PLGA was investigated in detail. The *in vitro* cytotoxic effects of the PLGA(UCNPs/DOX) nanocapsules were evaluated in H460 cancer cells.

## Results and Discussion

**Figure 1:**
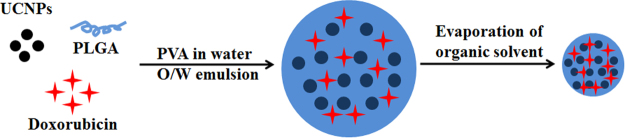
Schematic illustration of the preparation procedure of PLGA(UCNPs/DOX) nanocapsules.

The pH-responsive PLGA(UCNPs/DOX) nanocapsules were fabricated by using a facile and straightforward synthetic strategy, which is schematically illustrated in Figure 1. Hydrophobic NaYF_4_:Yb,Er@NaGdF_4_ NPs were synthesized in organic solvent according to our previous works^24–26,35^. The PLGA nanocapsules successfully encapsulating the inorganic nanocrystals as imaging agents and chemotherapeutic drug (DOX) were prepared by an oil-in-water (O/W) emulsion method and a subsequent solvent evaporation followed by polymer solidification at room temperature. Specifically, the hydrophobic DOX and NaYF_4_:Yb,Er@NaGdF_4_ NPs were incorporated into the hydrophobic domain of PLGA molecules via hydrophobic interaction, and the PLGA vesicles were then generated in the presence of poly(vinyl alcohol) (PVA) emulsifier. After the evaporation of the organic solvent in the emulsion, the PLGA(UCNPs/DOX) nanocapsules were collected using washing with deionized water and re-dispersed in phosphate buffer solution (PBS).

**Figure 2:**
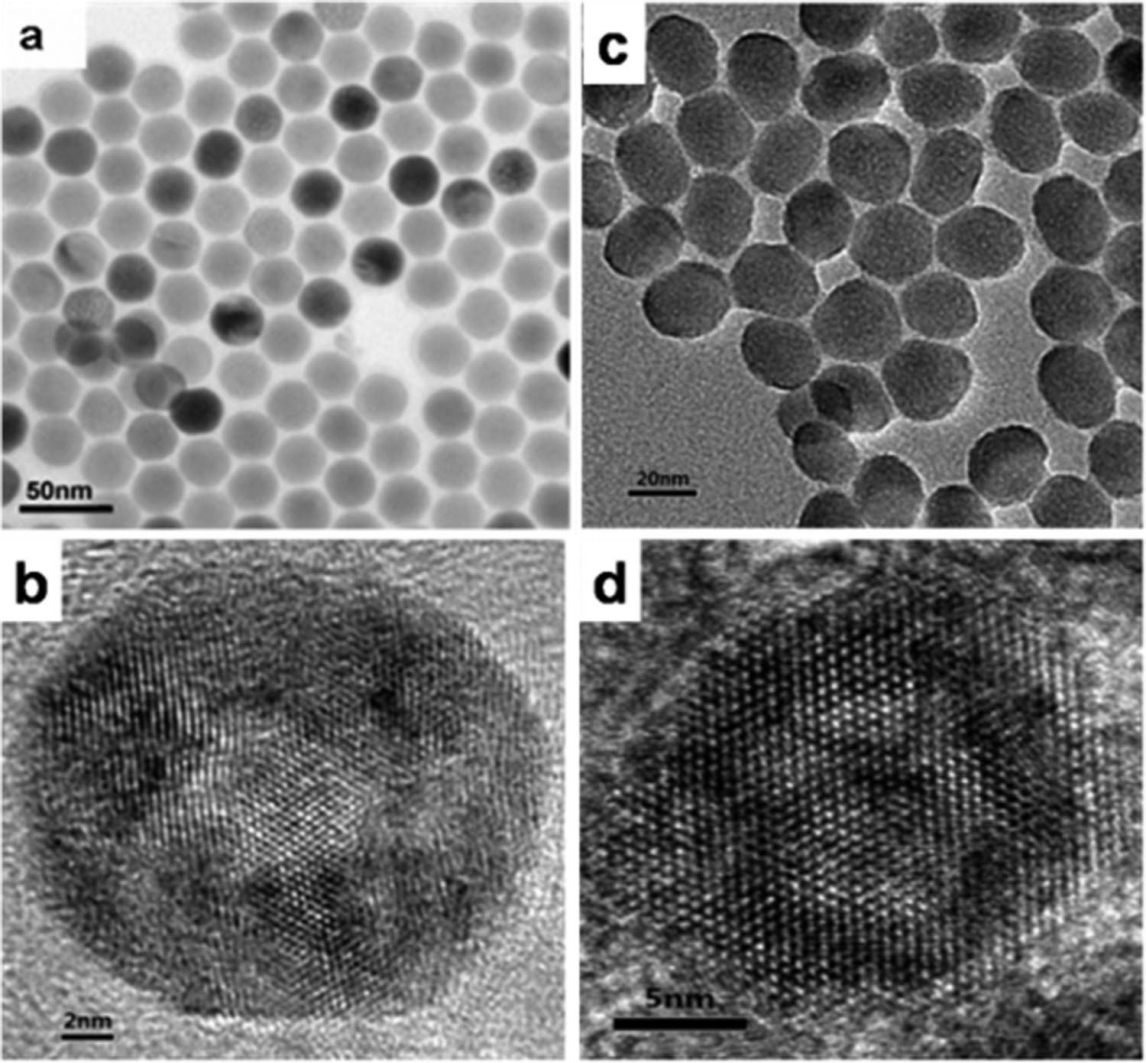
TEM and HRTEM images of NaYF_4_:Yb,Er NPs (**a**,**b**) and NaYF_4_:Yb,Er@NaGdF_4_ NPs (**c**,**d**).

The as-synthesized hydrophobic NaYF_4_:Yb,Er@NaGdF_4_ UCNPs were stabilized with oleic acid (OA), which were used as building blocks in the experiments. Figure S1 shows the XRD patters of NaYF_4_:Yb,Er nanocrystals. All intense peaks can be well indexed to hexagonal phase of NaYF_4_ (JCPDS No. 028–1192). In addition, no other phase or impurity peaks were detected, indicating the high purity of nanocrystals. The morphology and nanostructure of UCNPs were examined by transmission electron microscopy (TEM). According to TEM images of NaYF_4_:Yb, Er nanocrystals (Fig. 2a), one can observe that the NaYF_4_:Yb, Er nanocrystals consisted of well dispersed nanospheres with an average diameter of about 20 nm. High resolution TEM imaging of a single NaYF_4_:Yb, Er nanocrystal shown in Figure 2b reveals high quality lattice fringes attributing to hexagonal NaYF_4_. The energy dispersive X-ray spectroscopy (EDS) confirms the presence of yttrium (Y), ytterbium (Yb), erbium (Er), sodium (Na) and fluorine (F) in the NaYF_4_ nanocrystals (Fig. S2a). As shown in Figure 2c,d, the NaYF_4_:Yb,Er nanocrystals were successfully coated with NaGdF_4_ shell and the size of the core/shell NaYF_4_:Yb,Er@NaGdF_4_ UCNPs was change to be about 23 nm, which is larger than that of the NaYF_4_:Yb,Er nanocrystals. The morphology of NaYF_4_:Yb,Er@NaGdF_4_ UCNPs becomes approximately spherical from uniform one. The EDS results show that the Gd element exists in NaYF_4_:Yb,Er@NaGdF_4_ UCNPs, showing the successful coating of NaGdF_4_ on the NaYF_4_:Yb,Er nanocrystals (Fig. S2b).

**Figure 3:**
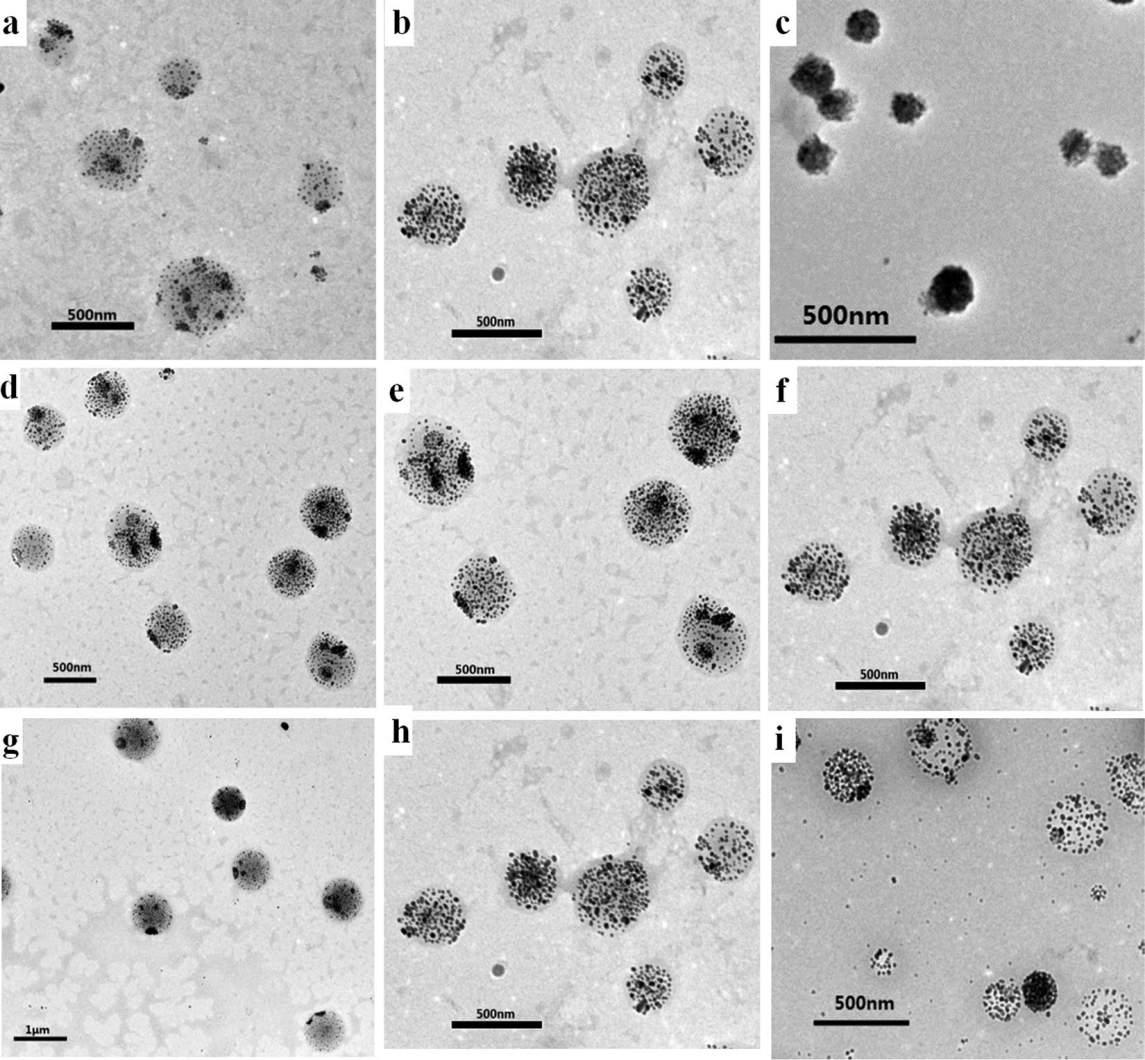
TEM images of PLGA(UCNPs/DOX) nanocapsules synthesized under different experiment conditions corresponding to Table 1. (**a**,**b**,**c**) PLGA-to-UCNPs mass ratios: 5:1 (**a**), 2:1 (**b**), 1:1 (**c**); (**d**,**e**,**f**) Stirring rate: 13000 rpm (**d**), 16000 rpm (**e**), 19000 rpm (**f**); (**g**,**h**,**i**) PVA concentration: 0.5 wt% (**g**), 1.0 wt% (h), 2.0 wt% (**i**).

Interestingly, we found that experimental parameters have a great influence on the size of the PLGA(UCNPs/DOX) nanocapsules. The effect of different experiment conditions on the average particle size of the PLGA(UCNPs/DOX) nanocapsules is shown in Table 1. In general, the PLGA-to-UCNPs mass ratios, the stirring rate of the emulsifying process and the PVA concentration have relatively large impact on the morphology of the samples. Figure 3 displays the representative TEM images of the corresponding samples prepared at different experimental conditions. The average size of the nanocapsules decreased as the decrease of PLGA-to-UCNPs mass ratios, the increase of the stirring rate of the emulsifying process and the PVA concentration. For the biological applications, we selected nanocapsules with smaller size and better dispersibility as the best samples. The optimum conditions are concluded as follow: the mass of PLGA and NaYF_4_:Yb,Er@NaGdF_4_ is 8.0 mg and 4.0 mg, respectively; the oil-to-water ratio is 0.1; the concentration of PVA is 2.0 wt% and the stirring rate of the emulsifying process is 19000 rpm. PLGA(UCNPs/DOX) nanocapsules that were prepared under the optimum preparation conditions were selected to use for the biomedical experiments.

**Table 1:** The effect of different experiment conditions on the size of PLGA(UCNPs/DOX) nanocapsules.

No.	PLGA:UCNPs	Stirring rate (rpm)	PVA concentration	Average size (nm)
1(a)	5:1	19000	1%	401
2(b,f,h)	2:1	19000	1%	315
3(c)	1:1	19000	1%	150
4(d)	2:1	13000	1%	553
5(e)	2:1	16000	1%	509
6(g)	2:1	19000	0.5%	766
7(i)	2:1	19000	2%	230

**Figure 4:**
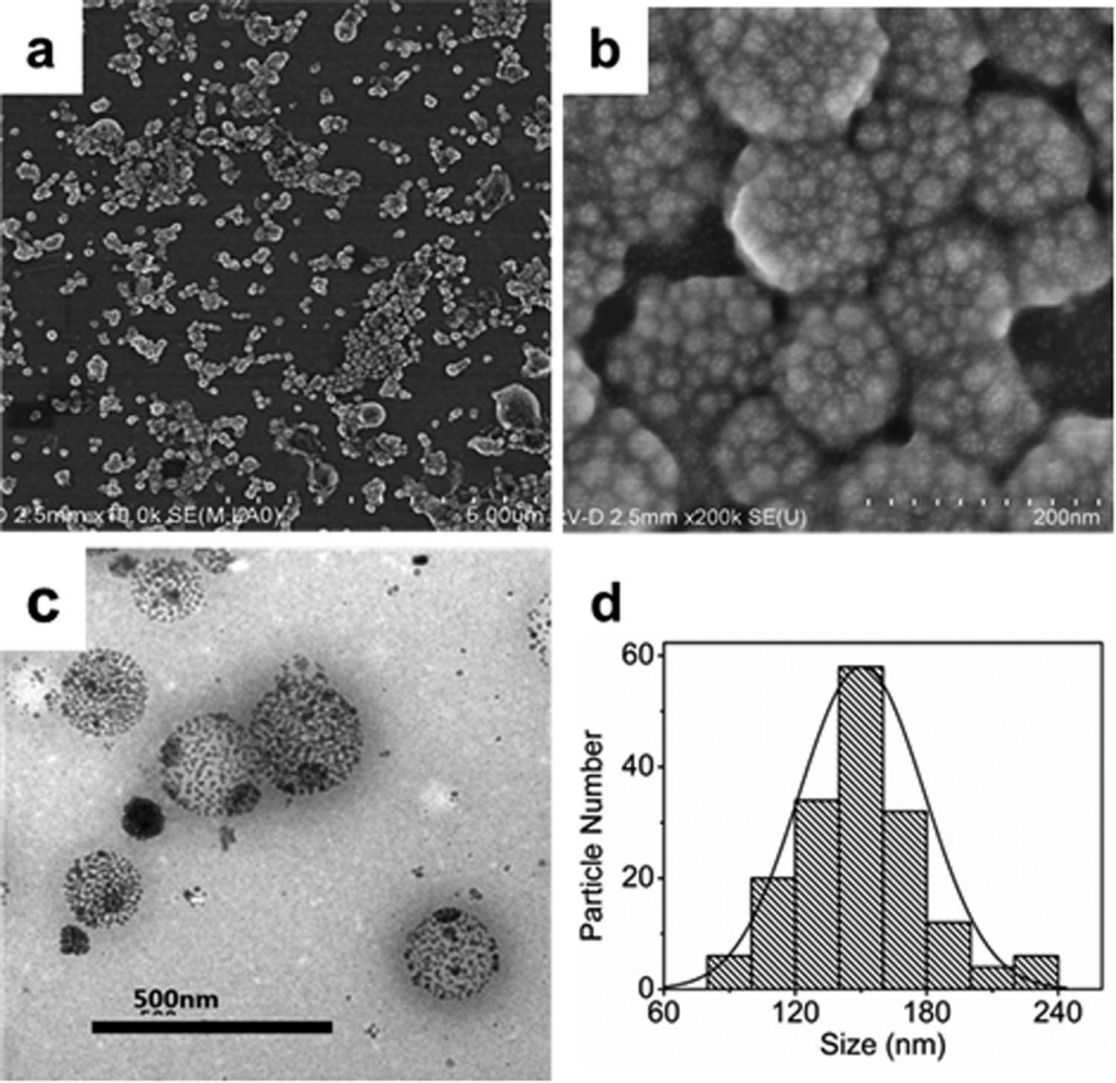
The SEM (**a**,**b**), TEM (**c**) images and the size distribution (**d**) of PLGA(UCNPs/DOX) nanocapsules synthesized under the optimized condition. PLGA: 8.0 mg; NaYF_4_:Yb,Er@NaGdF_4_: 4.0 mg; oil-to-water ratio is 0.1; the PVA concentration is 2.0 wt%; the stirring rate :19000 rpm.

As shown in Figure 4, the SEM and TEM images of the PLGA(UCNPs/DOX) nanocapsules prepared under optimum conditions demonstrate the spherical shape, smooth surface, and the average size of about 150 nm. The histogram distribution of particle sizes is displayed in Figure 4d. Furthermore, as shown in Figure 4c, the loading of these UCNPs in PLGA vesicles can be seen clearly and more than 250 UCNPs are encapsulated in the nanocapsules. Although the size of the nanocapsules is not very uniform, they share similar internal structures. The UCNPs embedded in organic polymer materials were compactly aggregated and distributed homogeneously within the whole volume. Moreover, a closer look at TEM images of the nanocapsules reveals that the gap distances between the nanocrystals are slightly larger as compared to the superparticles in our previous report^25^. The increase of interparticle distances would be contributed to the addition of the PLGA polymer that quenched the aggregation during the self-assembly of UCNPs.

**Figure 5:**
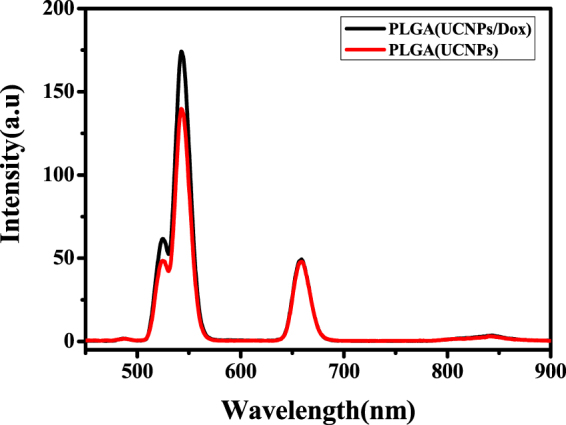
The upconversion luminescence spectra of PLGA(UCNPs) and PLGA(UCNPs/DOX) nanocapsules under 980 nm excitation.

The upconversion fluorescence spectra of NaYF_4_:Yb,Er NPs and NaYF_4_:Yb,Er@ NaGdF_4_ UCNPs under 980 nm excitation are displayed in Figure S3. Compared with NaYF_4_:Yb,Er NPs, NaYF_4_:Yb,Er@NaGdF_4_ UCNPs exhibit similar emissions except for the increase of intensity, suggesting that the NaGdF_4_ shell on the surface of UCNPs affect significantly the upconversion luminescence properties. The influence of core-shell structure on the luminescence properties of upconversion nanomaterials has been discussed in many literatures^36–40^. The upconversion fluorescence spectra of PLGA (UCNPs) and PLGA (UCNPs/DOX) nanocapsules under 980 nm laser excitation are also shown in Figure 5, which are similar with that of NaYF_4_:Yb,Er@NaGdF_4_ UCNPs. The upconversion fluorescence spectra of the PLGA (UCNPs/DOX) nanocapsules exhibit a green peak at about 520–540 nm and a red peak at about 654 nm, which are assigned to the transitions from ^2^H_11/2_ → ^4^I_15/2_, ^4^S_3/2_ → ^4^I_15/2_ and ^4^F_9/2_ → ^4^I_15/2_ of Er^3+^ ions, respectively. The upconversion fluorescence mechanism of Er^3+^, Yb^3+^ co-doped UCNPs is schematically illustrated in Figure S4. Yb^3+^ ions absorb initially a 980 nm NIR photon and subsequently transferred the energy to a nearby Er^3+^ ion, exciting Er^3+^ ion to the ^4^I_11/2_ level. Then a second 980 nm photon absorbed by the excited Yb^3+^ ion can populate Er^3+^ in the ^4^I_11/2_ level to the ^4^F_7/2_ level, afterward Er^3+^ will relax nonradiatively to the ^2^H_11/2_ and ^4^S_3/2_ levels. Finally, the transition from ^2^H_11/2_ → ^4^I_15/2_, ^4^S_3/2_ → ^4^I_15/2_ and ^4^F_9/2_ → ^4^I_15/2_ of Er^3+^ ions result in the green (520 nm, ^2^H_11/2_ → ^4^I_15/2_; 540 nm, ^4^S_3/2_ → ^4^I_15/2_) and red (654 nm, ^4^F_9/2_ → ^4^I_15/2_) emission, respectively. These results indicate that the upconversion fluorescence properties still retain after the UCNPs are incorporated into the nanocapsules.

**Figure 6:**
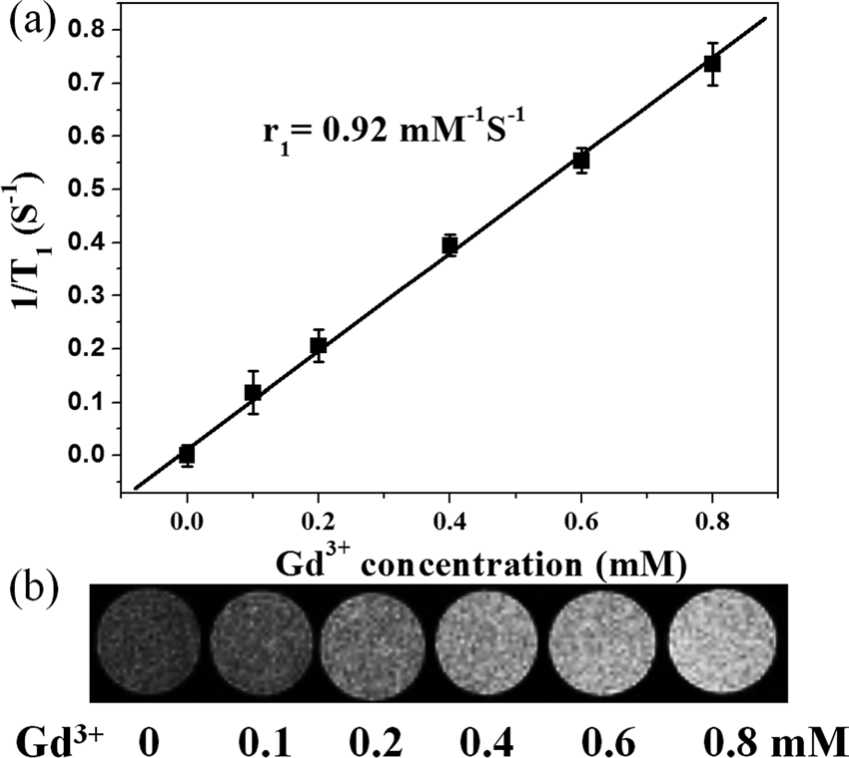
*T*_*1*_-weithted MRI contrast images of PLGA(UCNPs/DOX) nanocapsules with different concentrations.

Next, to evaluate the potential application of PLGA (UCNPs/DOX) nanocapsules in MRI, the proton longitudinal relaxation rates (1/*T*_1_) as function of Gd^3+^ concentration were determined using Bruker AVANCE 500WB spectrometer at 11.7 T, which shows a linear relationship (Fig. 6a). Furthermore, the signal intensity of *T*_1_-weighted MRI increased with increasing concentration of PLGA nanocapsules, demonstrating that Gd^3+^-containing UCNPs could be an effective *T*_1_-weighted MRI contrast agent (Fig. 6b). Based on the slope of the plot in Figure 6a the longitudinal relaxivity value (*r*_1_) was determined to be 0.92 mM^−1^S^−1^, smaller than that of most gadolinium chelates (Gd-DTPA, 4.1 mM^−1^S^−1^; Gd-DOTA, 3.6 mM^−1^S^−1^)^29^. This is likely to be attributed to the lack of strong interaction between neighboring water proton, as the Gd-contained UCNPs are both hydrophobic in nature and their surface still capped with OA.

**Figure 7:**
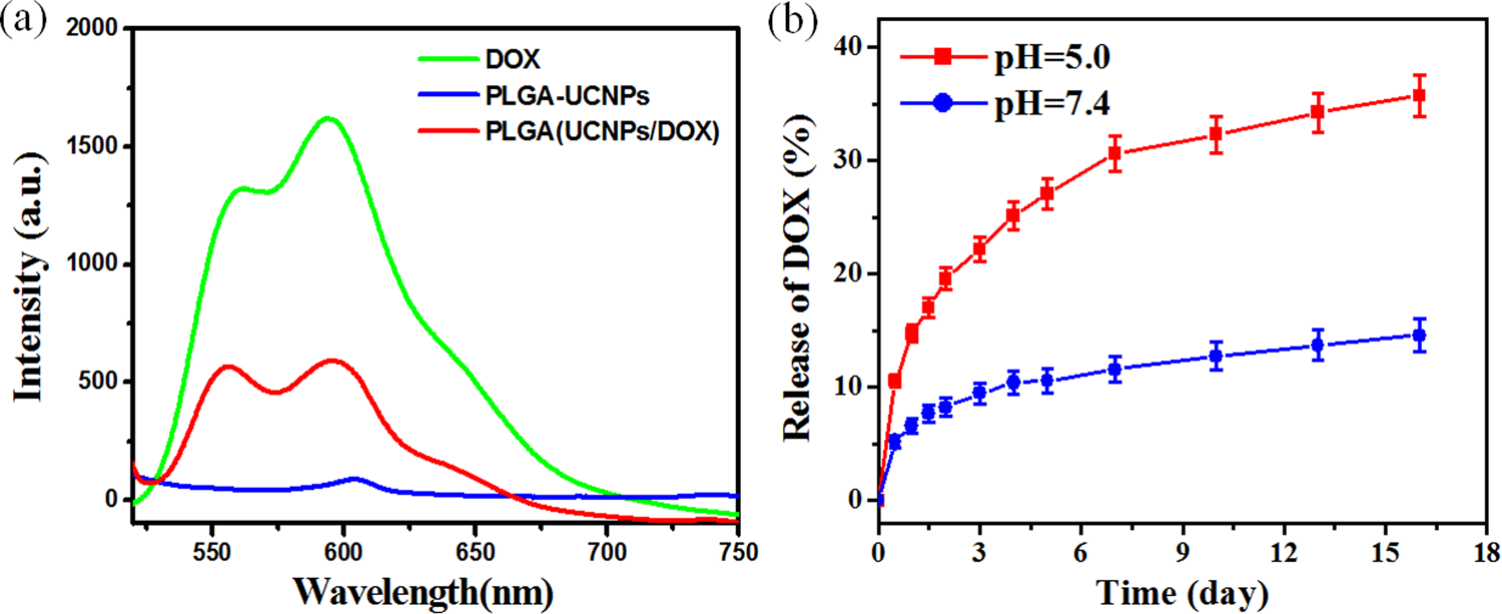
(**a**) Fluorescence spectra of DOX, PLGA(UCNPs) and PLGA(UCNPs/DOX) with the same DOX concentration (5.0 μg/ml) and UCNPs concentration under 490 nm excition. (**b**) Drug release curve of DOX from PLGA(UCNPs/DOX) nanocapsules in PBS buffer solution with different pH value. Error bars were based on triplicate samples.

It has been reported that many drug molecules with aromatic structure could be efficiently incorporated in biodegradable and biocompatible polymer as drug vehicles *via* hydrophobic interaction^16,17,41^. Herein, we selected a commonly used aromatic chemotherapy drug, DOX, as a model drug to examine the drug release of PLGA nanocapsules. To confirm DOX was encapsulated into the PLGA nanocapsules, we checked the fluorescence spectra of PLGA(UCNPs/DOX) nanocapsules under 490 nm excitation. As shown in Figure 7a, compared with free DOX, the same fluorescence characteristics were observed in the nanocapsules containing the same DOX concentration, apart from weakening the fluorescence intensity due to the concentration quenching. This data indicates that DOX can be successfully encapsulated into the PLGA nanocapsules with no impact on the fluorescence properties. The drug loading efficiency of the nanocapsules is crucial for the clinical application. Figure S5 shows the standard curve of DOX in the concentration range of 0.5–7.0 μg/mL. Based on a widely adapted method^10,16^, the loading efficiency was calculated using the UV-vis absorption spectra at 490 nm. Table S1 shows the DOX loading efficiency of the PLGA nanocapsules with different mass of DOX. It was measured that DOX loading efficiency increased with increasing the amount of added DOX up to 1000 μg. Furthermore, the loading efficiency was calculated up to 7.38% for the PLGA(UCNPs/DOX) nanocapsules. Therefore, the antitumor drug DOX can be effectively embedded in the PLGA nanocapsules to build up a theranostic platform.

The *in vitro* drug-releasing profiles of PLGA (UCNPs/DOX) nanocapsules under various environmental pH values are demonstrated in Figure 7b when dialyzing the PLGA (UCNPs/DOX) nanocapsules in pH 5.0 and 7.4 phosphate buffers at a temperature of 37 °C. The released DOX from nanocapsules was collected and then measured by fluorescence spectroscopy. It can be seen that the PLGA(UCNPs/DOX) nanocapsules reveal a sustained release profile and show a higher DOX release rate at pH 5.0 (mimicking microenvironment in endosomes and lysosomes) than those at pH 7.4 (physiological pH of blood stream). At pH 5.0, about 19.55 wt% of DOX was released from the nanocapsules at the first two days. However, the drug release observed in physiological pH 7.4 is as little as 8.26 wt%. At the sixteenth day, about 35.7 wt% and 14.58 wt% of released DOX were observed at pH 5.0 and 7.4, respectively. This phenomenon is due to a weak electrostatic interaction between DOX and PLGA matrix at low pH values. Meanwhile, the degradation of PLGA polymer at pH 5.0 was faster than that at pH 7.4, which may also contribute to the faster release of DOX under weak acidic pH environment^42^. The drug release studies indicate good stability of electrostatically bound drug molecules (PLGA-DOX system) in physiological pH and triggered release at acidic conditions. Therefore, our PLGA(UCNPs/DOX) nanocapsules are pH-responsive systems for DOX delivery and suitable for the specific treatment of solid tumors^41^.

**Figure 8:**
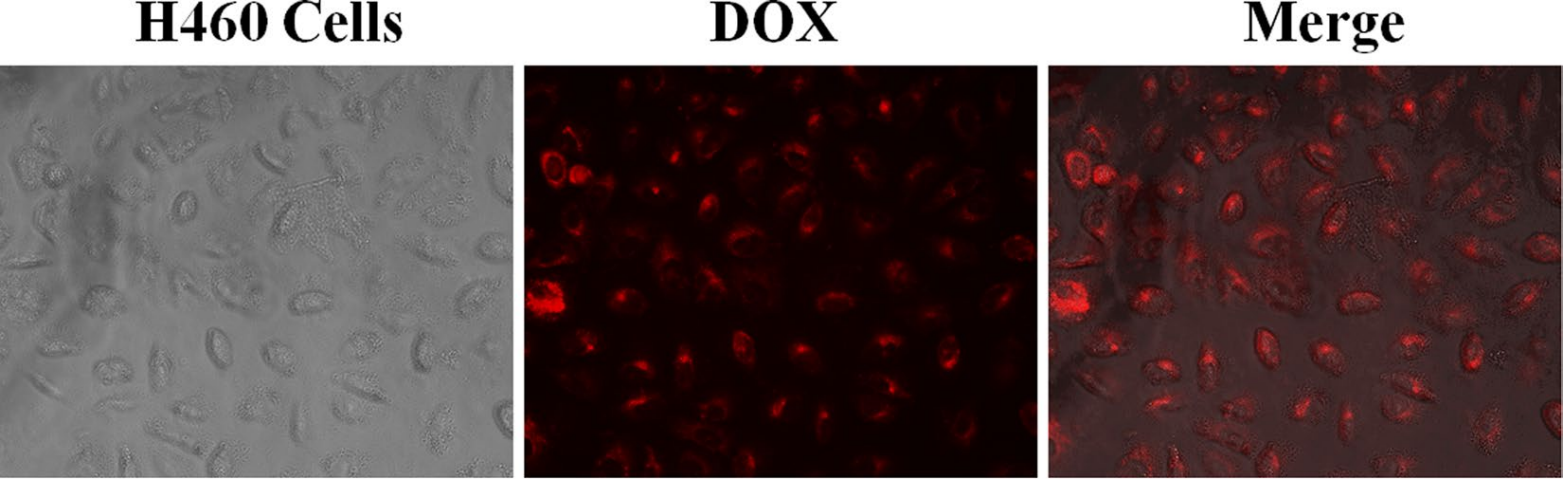
Optical microscope images (left), fluorescence image (middle) and merge image (right) of H460 cells after incubation with PLGA(UCNPs/DOX) nanocapsules for 4.0 h.

The results obtained from drug release studies inspired us to explore the cellular uptake and cytotoxicity of PLGA(UCNPs/DOX) nanocapsules on Human lung cancer cell lines (H460) since these are critical factors in evaluating the potential of new drug delivery system. The intercellular uptake of PLGA(UCNPs/DOX) nanocapsules was investigated using optical and fluorescence microscopy (*via* DOX fluorescence). As shown in Figure 8, it was revealed that the PLGA(UCNPs/DOX) nanocapsules were highly efficient at delivering DOX into cancer cells, where strong DOX fluorescence was observed inside cancer cells after 4 h incubation. Although free DOX could also enter and accumulate inside cells by diffusing, the PLGA(UCNP/DOX) nanocapsules were internalized mainly *via* endocytosis^43^. After cellular uptake, DOX was released from PLGA nanocapsules in acidic environment around endosome/lysosomes, where enough low pH (4.3) would trigger efficient DOX release (~ pH 5.0, Fig. 7b). Cellular uptake of PLGA(UCNP/DOX) nanocapsules were further verified using the upconversion fluorescence of UCNPs by employing a modified laser scanning confocal microscope. The PLGA(UCNP/DOX) nanocapsules showed a time dependent uptake in the H460 cancer cells, as seen in Figure S6. At 0.5 h post incubation, cellular uptake was apparent as demonstrated by a weak visible upconversion fluorescence, which appears to be localized in the cytoplasm. Moreover, an increase in the intercellular fluorescence intensity was observed in the H460 cells when increasing the incubation time to 4 h. Obviously, as time prolonging, more and more PLGA nanocapsules can enter the cancer cells. The above results confirm that the PLGA nanocapsules are highly efficient to deliver DOX into H460 cancer cells. Meanwhile, the released DOX from the nanocapsules in the cytoplasm pass through the nucleus membrane and eventually assembly in nucleus to kill the cell by causing conformation changes in the DNA^44^.

**Figure 9:**
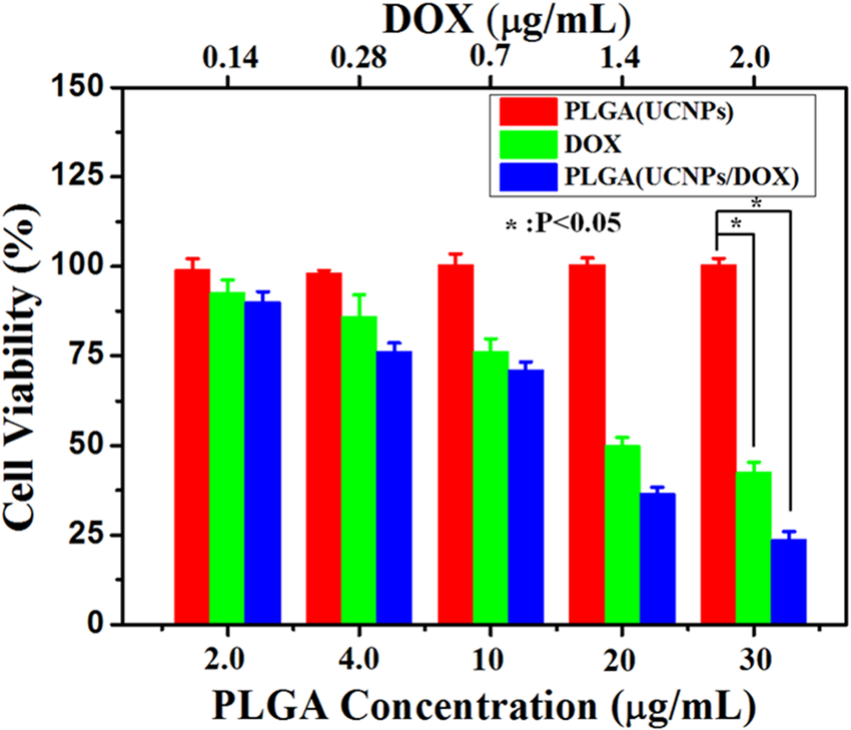
Relative viability of H460 cells incubated with different concertrations of PLGA(UCNPs) nanocapsules, PLGA(UCNPs/DOX) nanocapsules and DOX for 48 h *via* MTT assay (^*^P < 0.05). Error bars were based on triplicate samples.

To evaluate the pharmacological activity of the DOX-loaded nanocapsules, the *in vitro* cytotoxic effect of PLGA(UCNPs/DOX) on H460 cancer cells was assessed *via* MTT assay (MTT = 3-(4,5-dimethylthiazol-2-yl)-2,5-diphenyltetrazolium bromide)). Figure 9 shows the cell viabilities against free DOX, blank PLGA(UCNPs) and DOX-loaded PLGA(UCNPs/DOX) nanocapsules at different concentrations after incubation with H460 cells for 48 h. When incubated with PLGA(UCNPs) nanocapsules for 48 h, the cell viability was more than 90% with PLGA(UCNPs) concentration from 2.0 μg/mL up to 30 μg/mL, comparable to that of PLGA(UCNPs/DOX) nanocapsules at the same concentration. It was revealed that the blank PLGA(UCNPs) nanocapsules showed no obvious cytotoxic effect on cancer cells after 48 h treatment, even at the concentration as high as 30 μg/mL. This result demonstrates that the PLGA(UCNPs) nanocapsules are highly biocompatible. To demonstrate that the intracellular delivery of DOX is pharmacologically active, H460 cancer cells were treated with free DOX and PLGA(UCNPs/DOX) nanocapsules, respectively. When the free DOX concentration was set to be the same as that in the PLGA(UCNPs/DOX) nanocapsules, the cellular viability progressively decreased with increasing effective DOX concentration. As shown in Figure 9, after incubating with cells for 48 h, the free DOX and PLGA(UCNPs/DOX) nanocapsules exhibited noticeable cytotoxicity (P < 0.05). As the DOX concentration was increased from 0.14 μg/mL up to 2.0 μg/mL, the relative cell viability from about 90% decreased to about 30%. This result implies that both free DOX and PLGA(UCNPs/DOX) nanocapsules demonstrate dose-dependent cytotoxicity toward cancer cells and the cytotoxicity comes from the loaded DOX, not the PLGA molecules. Although free DOX exhibited a slightly higher cytotoxicity than the DOX-loaded nanocapsules (PLGA(UCNPs/DOX)) at the lower concentration, the DOX-loaded nanocapsules exhibited similar level of cytotoxicities on H460 cancer cells compared to free DOX when the concentration of DOX is up to 2.0 μg/mL. Therefore, free DOX is faster than the DOX-loaded nanocapsules by cellular uptake because the small DOX molecules could be diffused rapidly into cells whereas the nanocapsules must be endocytosed to enter the cancer cells. As the concentration increases, more and more DOX-loaded nanocapusles can be endocytosed to enter the cancer cells and then release DOX, which lead to the cancer cell death. In addition, solid tumors have a more acidic extracellular environment of pH < 7.0 than the normal tissues due to the hypxia-induced coordinated upregulation of glycolysis^45^. At the cellular level, the internalization of most nanoparticles will occur *via* endocytosis. After being engulfed by cells, normally the nanoparticles are trafficked into the early endosomes, then into the late endosomes/lysosomes, and finally fused with lysosomes. Both endosomes (pH 5.0–6.0) and lysosomes (pH 4.5–5.0) have an acidic microenvironment. In the present study, the pH-responsive PLGA(UCNPs/DOX) nanocapsules prefer to decompose and release drug at the acidic environment, which can effectively decrease the side effects and prolong the drug half-life for more effective, long lasting treatment. The released DOX molecules were located in the cell nucleus. It is well known that the cell nucleus is main target site of DOX and the DOX can attach to double-stranded DNA to form DNA adducts, thus inhibiting the activity of topoisomerase and inducing cell death (apoptosis)^46^. Moreover, an advantage in the fabrication of PLGA nanocapsules is simple one-step synthesis, where the DOX drug is loaded directly and conveniently in native form, eliminating any chemical modification and coupling steps that may alert the property and/or therapeutic efficacy of DOX drug. Therefore, the pH-responsive PLGA(UCNPs/DOX) nanocapsules may have good potential for cancer chemotherapy.

## Conclusion

In summary, we have successfully developed a multifunctional pH-responsive theranostic nanocapsules as drug delivery system for effective cancer chemotherapy, as well as for rapid, efficient and pH-triggered drug release using a simple and one-step emulsification method. The *in vitro* experiments showed that the PLGA(UCNPs/DOX) nanocapsules could operate as a multifunctional theranostic agents: they not only acted as contrast agents to enhance MRI and as excellent upconverted luminescence probe for cell imaging, but also exhibited an excellent cellular cytotoxic effect on H460 cells through pH-triggered DOX release at acidic environment. Overall, the biocompatible PLGA(UCNPs/DOX) nanocapsules as multifunctional theranostic platform has great potential for advancing biomedical application, including bioimaging, stimuli-responsive drug delivery nanocarries, and combinational or synergistic therapy together with other therapies.

## Methods

### Materials and reagents

Rare earth oxides Y_2_O_3_ (99.99%), Yb_2_O_3_ (99.99%), and Er_2_O_3_ (99.99%) were obtained from China Rare Earth Online Co. Ltd. Rare earth chlorides (LnCl_3_, Ln: Y, Gd, Yb, Er) were prepared by dissolving the corresponding oxides in hydrochloric acid and then evaporating the water completely. Ammonium fluoride (NH_4_F, 98%), sodium hydroxide, hydrochloric acid, ethanol, methanol, methylene chloride, triethylamine (TEA), and dimethyl sulfoxide (DMSO) were purchased from Sinopharm Chemical Reagent Co., Ltd (China). Oleic acid (OA, 90%), 1-octadecene (ODE, 90%), and 3-(4,5-dimethylthiazol-2-yl)-2,5-diphenyltetrazolium bromide (MTT) were purchased from Sigma-Aldrich. Anticancer drug doxorubicin hydrochloride (DOX, > 98%) was purchased from Shanghai Sangon Biotech Company (Shanghai China) and was deprotonated with addition of triethylamine to obtain the hydrophobic form. Carboxyl-terminated Poly(lactide-co-glycolide) (PLGA-COOH 50:50, Mw = 10000) was obtained from Jinan Daigang Bioengineer Company, China. Polyvinyl alcohol (PVA, Mw = 22000) was obtained from Kuraray CO. LTD, Japan. All reagents were used as received without further purification.

### Synthesis of NaYF_4_:Yb,Er@NaGdF_4_ Upconversion Nanoparticles (UCNPs)

The monodisperse NaYF_4_:Yb,Er nanocrystals were prepared according to a previously reported protocol with minor modification^24^. For the synthesis of NaYF_4_:Yb,Er@NaGdF_4_ core@shell nanocrystals, GdCl_3_ (1.0 mmol) was added in to a 50 mL flask containing 6.0 mL OA and 15 mL ODE. The mixture was slowly heated to 160 °C under argon atmosphere and maintained this temperature for 1 hour to obtain a homogeneous transparent yellow solution. The system was then cooled down to 80 °C, following the addition of 1.0 mmol as-prepared NaYF_4_:Yb,Er nanocrystals in 6.0 mL of cyclohexane. After the remove of cyclohexane, a 10 mL methanol solution containing 4.0 mmol of NH_4_F and 2.5 mmol of NaOH was added and the system was stirred at 50 °C for 30 min. After methanol evaporated, the system was then heated to 300 °C for 1.5 h under argon atmosphere, and then cooled down to room temperature. The nanocrystals were precipitated with ethanol and collected by centrifugation. The obtained nanocrystals redispersed in dichloromethane.

### Synthesis of PLGA (UCNPs/DOX) nanocapsules

Firstly, 1.0 mg DOX was dissolved in 50 μL water under oscillating conditions to form red solution. Then, 2.0 μL of thiethylamine was added under shock conditions for 2 h to extract DOX. Secondly, 1.0 mL dichloromethane solution including 8.0 mg PLGA, 4.0 mg NaYF_4_:Yb, Er@NaGdF_4_ and 1.0 mg extracted DOX was added to 10 mL PVA (2 wt%) aqueous solution. Thirdly, the solution system was vigorous stirring for 10 min to form microemulsion system. Finally, the emulsions were continuously stirred at 600 rpm at room temperature for 6 h to evaporate the organic solvent. The final products were washed with deionized water three times to remove free nanocrystals, giving a typical yield of about 70%. For comparison, the product of PLGA (UCNPs) nanocapsules without DOX was also prepared under similar experimental conditions.

### MRI Measurements

The MRI measurements were performed in an 11.7 T micro 2.5 micro imaging system (Bruker, Germany). The different amount of the obtained PLGA (UCNPs/DOX) nanocapsules were dispersed in 1.2 mL agarose aqueous solution and then loaded into the microtubes for MRI measurements. The final Gd^3+^ concentration were 0 mM, 0.1 mM, 0.2 mM, 0.4 mM, 0.6 mM and 0.8 mM, respectively. The measurement parameters are as follows: repetition time (TR) = 300 ms, echo time (TE) = 4.5 ms, imaging matrix = 128 × 128, slice thickness = 1.2 mm, field of view (FOV) = 2.0 × 2.0 cm, and number of averages (NA) = 2.

### Standard curve of DOX

A suitable quantity of DOX was dissolved in water by oscillation. Then, a series of different concentrations of DOX aqueous solution were prepared (0.5 μg/mL, 1.0 μg/mLl, 3.0 μg/mL, 5.0 μg/mL, 7.0 μg/mL). The UV-vis absorption of different concentrations of DOX solution was measured (A_ab_ = 490 nm). Finally, the standard curve of DOX was determined through the curve fitting of the absorption *vs* the DOX concentration (Figure S5). The standard curve showed a good linear relationship in the range of concentration of 0.5~7.0 μg/ml.

### DOX Loading and Release

To measure the loading capacities of PLGA (UCNPs/DOX) nanocapsules, the supernatant solution was collected after centrifugation of the PLGA (UCNPs/DOX) nanocapsules. The absorption of DOX molecules in the supernatant solution was examined and the concentration of DOX in the supernatant was calculated by comparing the standard curve of DOX. The percentages of DOX remaining in the PLGA (UCNPs/DOX) nanocapsules were calculated according to the following equation:

$${\rm{Loading}}\,{\rm{efficiency}}\,( \% )=({{\rm{W}}}_{{\rm{0}}}-{{\rm{W}}}_{{\rm{s}}}){/W}_{{\rm{0}}}\times \mathrm{100} \% $$

where W_0_ and W_s_ represent the initial DOX mass and the DOX mass in the supernatants, respectively.

For the cumulative DOX release studies in PBS buffer solutions (pH 5.0, and 7.4) with the same NaCl concentration of 0.15 M, the PLGA (UCNPs/DOX) nanocapsules were dispersed in 5.0 mL of buffer solution and then transferred into a dialysis bag. Then it was kept in buffer solution and gently shaken at 37 °C. At selected timed intervals, 1.0 mL of solution was withdrawn and analyzed by UV-vis absorption. To retain a constant volume, 1.0 mL of fresh buffer was added after each sampling.

### *In vitro* cytotoxicity of PLGA (UCNPs/DOX) nanocapsules

*In vitro* cytotoxicity of the PLGA (UCNPs/DOX) nanocapsules was assessed against Human lung cancer (H460) cells based on the MTT assay. H460 cells were cultured in RPMI 1640 growth medium complemented with 10% fetal bovine serum (FBS), streptomycin at 100 μg/mL, and penicillin at 100 units/mL. The cells were maintained at 37 °C in a humidified atmosphere of 5% CO_2_ in air. The assay was performed in triplicate with the same manner. Briefly, H460 cells were seeded into 96-well plates at a density of 1 × 10^4^ cells per wells in 100 μL of media. After overnight growth, the cells were then incubated at various concentrations of DOX, PLGA (UCNPs) and PLGA (UCNPs/DOX) nanoclusters (2.0, 4.0, 10, 20, 30 µg/mL) for 48 h. The material contents were calculated according to the concentration of DOX. That is, the free DOX concentration was the same as the DOX concentration in PLGA (UCNPs/DOX) nanoclusters and the PLGA (UCNPs) concentration was the same as the PLGA (UCNPs) concentration in the PLGA (UCNPs/DOX) nanocapsules. After being incubated for 48 h, the 10 μL MTT solution (5 mg/mL) was then added each well and the cells were further incubated for 4 h at 37 °C. After the MTT solution was removed, 150 μL of dimethyl sulphoxide (DMSO) was added to each well and the plate was gently shaken for 10 min to dissolve the precipitated violet crystals. The optical density (OD) was measured at 490 nm using microplate reader (Perkin Elmer, Victor × 4). Cell viability was evaluated as a percentage compared to control cells.

### Characterization

The sizes and morphologies of NaYF4:Yb,Er, NaYF_4_:Yb,Er@NaGdF_4_ UCNPs and PLGA (UCNPs/DOX) nanocapsules were examined by using a Hitachi S-4800 scanning electron microscope (SEM) equipped with an energy-dispersive X-ray spectrometer and a FEI Tecnai G2-F20 transmission electron microscope (TEM) at an accelerating voltage of 200 kV. The UV-vis absorption spectra were acquired by a Perkin Elmer Lambda-25 UV-vis spectrometer. The fluorescence spectra were recorded using a Hitachi F-4600 fluorescence spectrophotometer. For upconversion fluorescence spectra, a CW 980 nm semiconductor laser diode (BWT Beijing Ltd, China) was used as the excitation source to replace an internal Xe lamp. Inductively coupled plasma atomic emission spectroscopy (ICP-AES) (Agilent 5100) was used to analyze the element Gd concentrations in the PLGA (UCNPs/DOX).

## Electronic supplementary material

Supplementary information
