# Supplementary material for: Fabrication of pH-responsive PLGA(UCNPs/DOX) nanocapsules with upconversion luminescence for drug delivery
Source: Sci Rep. 2017 Dec 21;7:18014. doi: 10.1038/s41598-017-16948-4 (PMC5740179; doi:10.1038/s41598-017-16948-4)
Supplement: Supplementary file 1 — Supplementary information [file 41598_2017_16948_MOESM1_ESM.pdf]

Supplementary information

Construction of pH-responsive PLGA(UCNPs/DOX) nanocapsules with upconversion luminescence for drug delivery

Junwei Zhao[1,2], Hui Yang[2], Jili Li[1], Yujiang Wang[1] &Xin Wang[2,*]

[1]Materials Science and Engineering School, Luoyang Institute of Science and Technology, Luoyang, 471023, P. R. China.

[2]Suzhou Key Laboratory of Nanobiomedicine, Division of Nanobiomedicine, Suzhou Institute of Nano-Tech & Nano-Bionics, Chinese Academy of Sciences, Suzhou, 215123, P. R. China.

Correspondence and requests for materials should be addressed to X.W. (email: xwang2008@sinano.ac.cn)

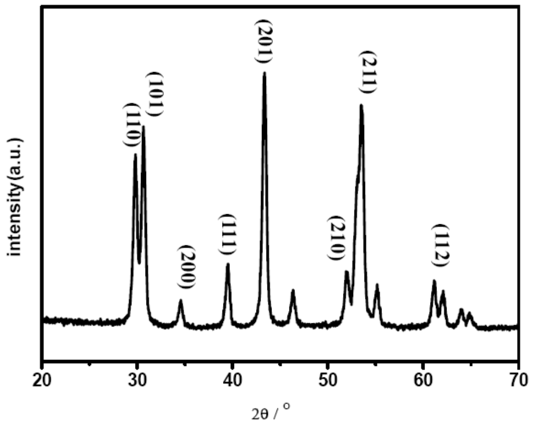

Figure S1. The XRD patters of $NaYF_4$:Yb,Er NPs.

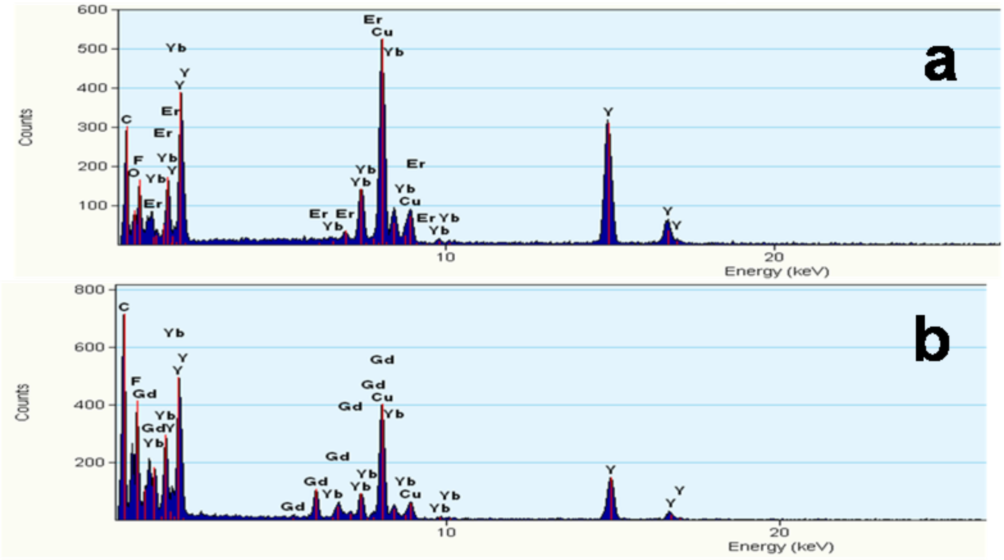

Figure S2. The energy dispersive X-ray spectra of $NaYF_4$:Yb,Er NPs (a)and $NaYF_4$:Yb,Er@$NaGdF_4$ NPs(b).

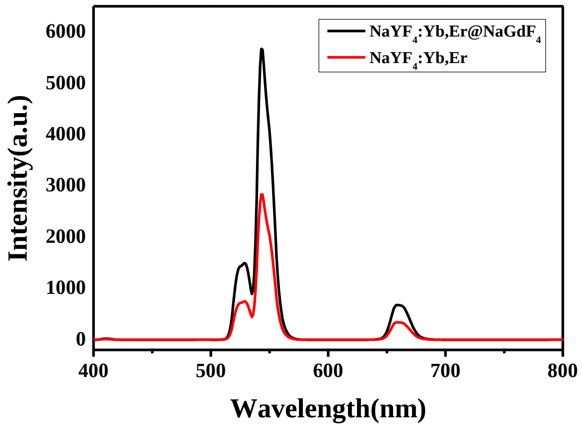

Figure S3. The upconversion luminescence spectra of $NaYF_4$:Yb,Er nanocrystals and $NaYF_4$:Yb,Er@ $NaGdF_4$ UCNPs under 980 nm excitation.

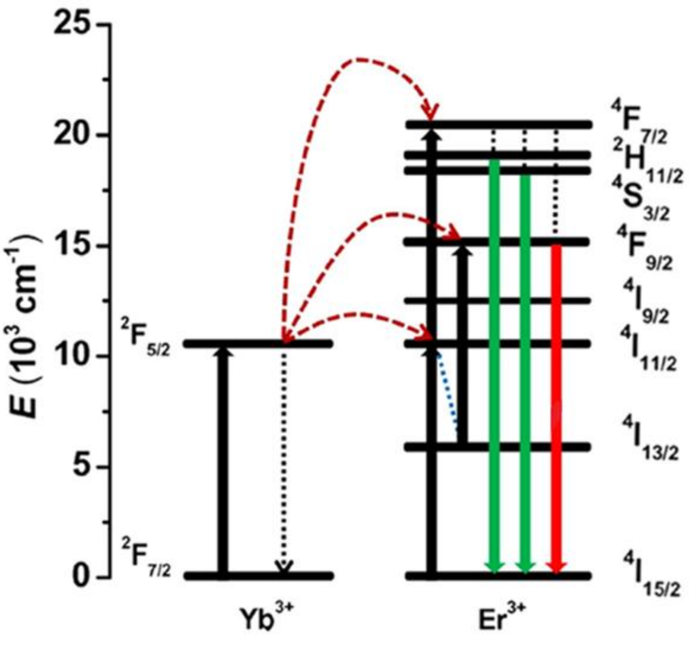

Figure S4. Schematic energy levels of Yb^{3+} and Er^{3+} and the UC luminescence process excited by 980 nm light.

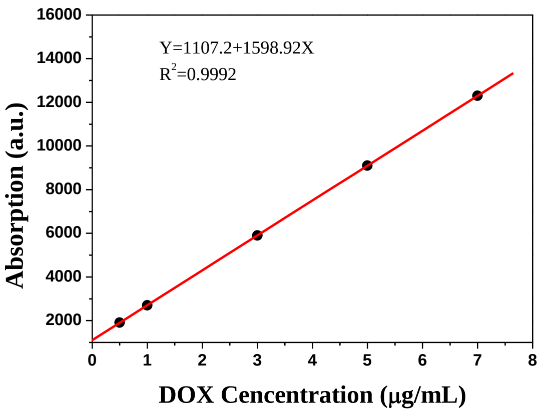

Figure S5. Standard curve of DOX.

Table S1. Drug loading efficiency of the drug-carriers with quantity change of DOX.

Added DOX	Loading efficiency (%) (i)	Loading efficiency (%) (ii)	Loading efficiency (%) (iii)	Average loading efficiency (%)
300 μg	1.21%	1.21%	1.21%	1.21%
500 μg	3.56%	3.55%	3.57%	3.56%
1000 μg	7.37%	7.23%	7.54%	7.38%

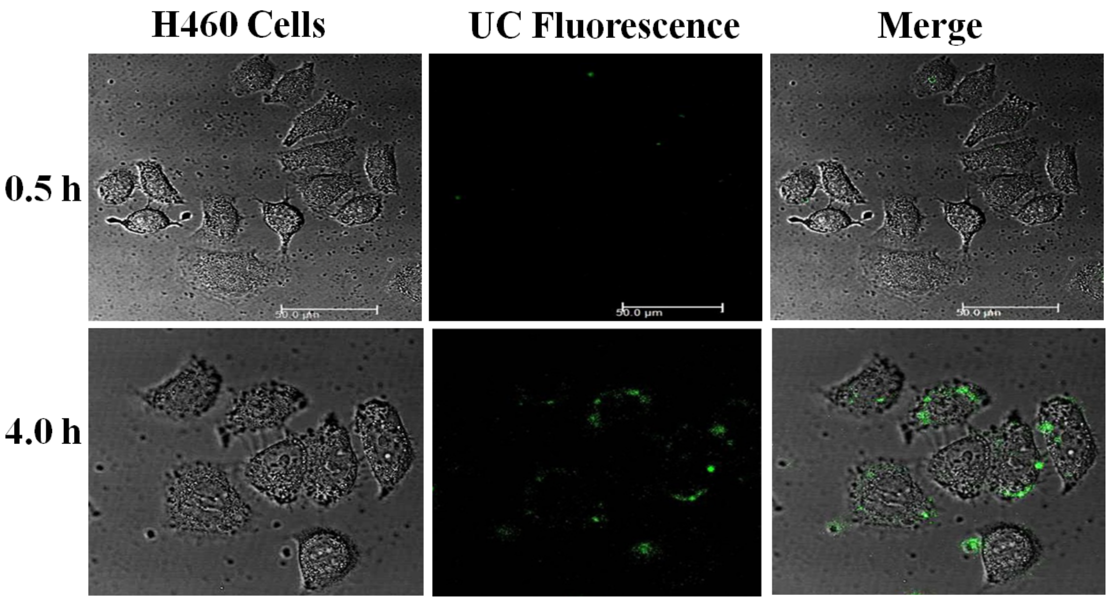

Figure S6. Confocal upconversion fluorescence microscope images of H460 cancer cells under excitation of 980 nm laser after incubation with PLGA(UCNPs/DOX) nanocapsules for 0.5 and 4.0 h, respectively.
